# Challenges Associated with Byproducts Valorization—Comparison Study of Safety Parameters of Ultrasonicated and Fermented Plant-Based Byproducts

**DOI:** 10.3390/foods9050614

**Published:** 2020-05-11

**Authors:** Elena Bartkiene, Vadims Bartkevics, Iveta Pugajeva, Anastasija Borisova, Egle Zokaityte, Vita Lele, Vytaute Sakiene, Paulina Zavistanaviciute, Dovile Klupsaite, Daiva Zadeike, Fatih Özogul, Grazina Juodeikiene

**Affiliations:** 1 Institute of Animal Rearing Technologies, Lithuanian University of Health Sciences, Tilzes g. 18, LT-47181 Kaunas, Lithuania; egle.zokaityte@lsmuni.lt (E.Z.); vita.lele@lsmuni.lt (V.L.); vytaute.sakiene@lsmuni.lt (V.S.); paulina.zavistanaviciute@lsmuni.lt (P.Z.); dovile.klupsaite@lsmuni.lt (D.K.); 2 Department of Food Safety and Quality, Lithuanian University of Health Sciences, Tilzes g. 18, LT-47181 Kaunas, Lithuania; 3 Centre of Food Chemistry, University of Latvia, Jelgavas iela 1, LV-1004 Riga, Latvia; vadims.bartkevics@bior.lv; 4 Institute of Food Safety, Animal Health and Environment BIOR, Lejupes iela 3, LV-1076 Riga, Latvia; iveta.pugajeva@bior.lv (I.P.); anastasija.borisova@bior.lv (A.B.); 5 Department of Food Science and Technology, Kaunas University of Technology, Radvilenu Rd. 19, LT-50254 Kaunas, Lithuania; daiva.zadeike@ktu.lt (D.Z.); grazina.juodeikiene@ktu.lt (G.J.); 6 Department of Seafood Processing Technology, The University of Cukurova, Balcali, Saricam, 01330 Adana, Turkey; fozogul@cu.edu.tr

**Keywords:** processing byproducts, press cakes, mycotoxins, biogenic amines, fermentation, ultrasonication

## Abstract

In order to promote the efficient use of byproducts from the production of plant-based beverages, which still contain a large amount of nutritional and functional compounds, microbiological and chemical safety characteristics should be evaluated and, if needed, improved. Many challenges are associated with byproducts valorization, and the most important ones, which should be taken into account at the further steps of valorization, are biological and chemical safety. For safety improving, several technological treatments (biological, physical etc.) can be used. In this study, the influence of low-frequency ultrasonication (US) and fermentation with *Lactobacillus casei* LUHS210 strain, as physical and biotechnological treatments, on the safety characteristics of the byproducts (BYs) from the processing of rice, soy, almond, coconut, and oat drinks was compared. Ultrasonication, as well as fermentation, effectively improved the microbiological safety of BYs. Ultrasonication and fermentation reduced the concentration of deoxynivalenol, on average, by 24% only in soy BYs. After fermentation, 15-acetyldeoxynivalenol was formed in all samples (<12 µg kg^−1^), except for soy BYs. The lowest total biogenic amines content was found in fermented rice BYs and ultrasonicated coconut BYs. When comparing untreated and fermented BYs, significant changes in macro- and micro-elements content were found. Ultrasonication at 37 kHz did not significantly influence the concentrations of macro- and micro-elements, while fermentation affected most of the essential micro-elements. Consequently, while ultrasonication and fermentation can enhance the safety of BYs, the specific effects must be taken into account on biogenic amines, mycotoxins, and micro and macro elements.

## 1. Introduction

Many different raw materials of plant origin, including soy, rice, almond, coconut, and oat, are used to produce plant-based beverages, since these drinks are desirable alternatives to cow’s milk, including for individuals with specific health disorders [1]. In the United States, plant-based beverages make up 9.3% of the total milk market [2] and is expected to grow at a current annual growth rate (CAGR) of over 14% during 2018–2024 worldwide [3]. Among all nondairy milk beverages, soya milk dominates in the global market and is the most preferable choice by consumers. In the European Union, soybeans production reached 8.77 million tons in 2014 [4], and approximately 13.5 million tons are imported each year [5]. The global rice milk market is also expected to register a CAGR of above 15% (during 2018 and 2023), while almond and coconut milk—14.3% by 2025 and 14.61% until 2023, respectively [3]. The general manufacturing procedure for plant-based drinks is based on the raw stock using water mixture pressing. After this technological treatment, the solid phase left is called a press cake [6]. For example, 50% of all soy-based food products is press cake (okara), which is usually underutilized and disposed of as low-grade feed or waste [5]. However, the press cake still contains a large amount of nutritional and functional compounds that may be valuable for food or the animal feed industry [7]. Plant byproducts (BYs) have a potential to diversify population diet as a source of minerals; thus, it is important to characterize changes of mineral content during valorization [8]. Minerals are involved in a wide range of physiological functions in body and mineral deficiency can cause a variety of health problems [9]. The different techniques of food processing (fermentation, ultrasonication, and other types of treatment) can lead to changes of the concentration of these minerals. Several studies about fermentation with lactic acid bacteria (LAB) have been published, as such treatment provides a promising approach for improving the biosafety and functional properties of fermentable substrates [10,11,12].

Meanwhile, to promote efficient use of press cake from the production of plant-based beverages, the safety of these press cake should be evaluated, and the identification of their microbial diversity, as well as chemical composition, should be carried out during various types of processing. The most promising techniques to be applied for microbial decontamination, disaggregation, and functionalization of press cake materials could be ultrasonication, as well as fermentation with starter cultures selected for their antimicrobial/antifungal properties [13]. Both fermentation and ultrasonication can be promising tools for enhancing the biosafety of press cake materials; however, the mechanisms of bacterial inactivation by microbial starters and ultrasonication are different.

The effects of ultrasonication on bacteria inactivation are mainly due to cavitation phenomena [14]. The relative stiffness and thickness of bacterial cell walls are considered to be the most important factors for effective microbial decontamination during ultrasonication. Bacteria with thin, stiff cell walls are more susceptible to rupture when subjected to ultrasound waves, whereas those with thicker, more flexible cell walls can better resist the mechanical effects due to cavitation. Also, ultrasonication has been described as an effective treatment for the degradation of several mycotoxins in aqueous solutions and food matrices [15,16]. However, the earlier studies did not analyze emerging and masking mycotoxins and also did not identify metabolites that can be formed after ultrasonication. It should be mentioned that ultrasonication can lead to degradation of proteins and other food matrix compounds that can be desirable [17,18]. Ultrasonication has been applied in food processing for various purposes [19], but the changes in content of mycotoxins and minerals, as well as the formation of biogenic amines (BAs) after ultrasonication, have not been sufficiently studied.

The properties of LAB to inhibit pathogenic strains are explained by their antagonistic systems excreted by starter cultures, and our previous studies showed that the *Lactobacillus casei* LUHS210, used in this study for the treatment of press cakes, inhibits various pathogenic and opportunistic microorganisms [12]. Also, during fermentation, some of the microbial starters excrete enzymes that can degrade mycotoxins into non-toxic compounds [20]. Fermentation with LAB strains typically provides beneficial effects but can also lead to decarboxylation of amino acids and the formation of harmful BAs [21]. Data are available about the formation of BAs and changes of mycotoxin content during fermentation [22,23,24,25,26]. In contrast, the concentration of BAs in fermented plant-based foods is not controlled, despite the fact that it is very important to evaluate BA formation during food processing [27,28].

Therefore, in this study, the influence of low-frequency ultrasonication (US) and fermentation with *Lactobacillus casei* LUHS210 strain as physical and biotechnological treatments on safety characteristics of byproducts (BYs) from the processing of rice, soy, almond, coconut, and oat drinks was compared.

## 2. Materials and Methods

### 2.1. Samples

Processing byproducts (RPC—rice press cake; SPC—soy press cake; APC—almond press cake; CPC—coconut press cake; OPC—oat press cake) were obtained from a European company producing plant-based drinks in 2018. Press cakes were stored in airtight containers at −18 °C until used for analyzes.

### 2.2. Microorganism for Fermentation

The *L. casei* LUHS210 was obtained from the Department of Food Safety and Quality at the Lithuanian University of Health Sciences (Kaunas, Lithuania). From previous studies, it was known that the LUS210 strain inhibited various pathogenic strains [12]. The LUHS210 strain, before an experiment, was stored at −80 °C (Microbank system, Pro-Lab Diagnostics, Birkenhead, UK) and multiplied in MRS (Man-Rogosa-Sharpe, CM 0359, Oxoid Ltd., Hampshire, UK), broth at 30 ± 2 °C for 48 h before their use for the fermentation of processing byproducts.

### 2.3. Chemicals

Sodium hydroxide, sodium chloride, formic acid, nitric acid, dansyl chloride, perchloric acid, sodium bicarbonate, acetonitrile (HPLC grade), ammonium acetate, sodium citrate tribasic dihydrate, and sodium citrate dibasic sesquihydrate were obtained from Sigma-Aldrich (St. Louis, MO, USA). Ethanol and methanol (HPLC grade) were from FarmaBalt (Riga, Latvia). Nitric acid (≥69.0%), hydrogen peroxide, (30% *w*/*w*, extra pure), and multielement standard solution V were obtained from Sigma-Aldrich (Lyon, France). Tryptamine hydrochloride, 1,4-diaminobutane dihydrochloride, 2-phenylethylamine hydrochloride, histamine dihydrochloride, cadaverine dihydrochloride, 1,7-diaminoheptane, tyramine hydrochloride, and spermidine phosphate salt hexahydrate were obtained from Sigma-Aldrich (St. Louis, MO, USA). Spermine diphosphate hexahydrate was from TCI Europe (Tokyo, Japan). The standards of the mycotoxins (15-acetoxyscirpenol, 15-acetyldeoxynivalenol, 17-dimethylaminoethylamino-17-demetoxy-geldanamycin, 17-(allyamino)–17-demetoxygeldanamycin, aflatoxin B1, aflatoxin B2, aflatoxin G1, aflatoxin G2, aflatoxin M1, aflatoxin Ro (aflatoxicol), altenuene, altertoxin I, alternariol monomethyl ether, ansamitocin P3, alternariol, apicidin, bafilomycin A1, bafilomycin B1, beauvericin, brefeldin A, cytochalasin A, cytochalasin B, cytochalasin C, cytochalasin D, cytochalasin E, cytochalasin H, cytochalasin J, cerulenin, chaetoglobusin A, chaetocin, curvularin, citreoviridin, 10,11–dehydrocurvularin, deoxynivalenol-3-glucoside, deoxynivalenol, destruxin A, destruxin B, dihydrochalasin B, enniatin A, enniatin A1, enniatin B, enniatin B1, fusaric acid, fumonisin B1, fumonisin B2, fumonisin B3, fusaric acid, fusarenon-X, gliotoxin, helvolic acid (fumagicin), HT-2 toxin, meleagrin, myriocin, neosolaniol, ochratoxin A, ochratoxin B, penicillic acid, penitrem A, paxilline, sterigmatocystin, wortmannin, roquerfortine-C, stachybotrylam, T-2 toxin, T-2 triol, T-2 tetraol, tentoxin, verruculogen, zearalenone), along with the certificates of proof of the contents of the compounds, were purchased from Romer Labs Biopure (Tulln, Austria). Mycotoxin standard substances were dissolved in acetonitrile to prepare stock solutions with a concentration of 1 mg/mL. The solutions were stored at −20 °C for a limited period of not more than six months, in accordance with the manufacturer’s recommendations. All reagents were of analytical grade.

### 2.4. Fermentation and Ultrasonication of Processing Byproducts

The processing byproducts, water, and a suspension of LUHS210 strain (3% from dry matter of the byproduct mass) containing 8.9 log_10_ CFU/mL, were fermented at 30 ± 2 °C for 48 h. For 100 g of processing byproduct, 60 mL water was used. The final moisture content of processing byproducts was on average 60%. The moisture content was determined according to the method described in reference [29].

Ultrasonication of byproducts was performed at 37 kHz frequency, using 100 W power level. The equipment employed was an ultrasonication processor (PROCLEAN 3.0DSP, Ulsonix, Berlin, Germany). Each 250 g sample of byproduct was processed for 30 min. The moisture content of processing byproducts was on average 60 ± 3%.

### 2.5. Acidity Characteristics of the Fermented Byproducts

The pH was measured using a pH electrode (PP-15; Sartorius, Goettingen, Germany). The total titratable acidity (TTA) was evaluated for a 10 g sample of byproduct mixed with 90 mL of water, and the results were expressed in mL of 0.1 mol/L NaOH solution required to achieve a pH value of 8.2. For L−(+) and D−(−) lactic acid isomers concentration evaluation, a specific Megazyme assay kit (Megazyme Int., Bray, Ireland) was used.

### 2.6. Microbiological Analysis of Processing Byproducts

The microbiological analysis of samples included the determination of LAB, total bacteria (TBC), enterobacteria (TEC), and mold/yeast (M/Y) counts. Microbiological analysis in details is given by Bartkiene et al. [11].

### 2.7. High-Performance Liquid Chromatography Coupled to Time of Flight High-Resolution Mass Spectrometry (HPLC-TOF-HRMS) for Mycotoxin Analysis

The following mycotoxins in byproducts were analyzed: 15-acetoxyscirpenol, 15-acetyldeoxynivalenol (15-AcDON), 17-dimethylaminoethylamino-17-demetoxygeldanamycin, 17-(allyamino)-17-demetoxygeldanamycin, aflatoxin B1 (AFB1), aflatoxin B2, aflatoxin G1, aflatoxin G2, aflatoxin M1, aflatoxin Ro (aflatoxicol), altenuene (ALT), altertoxin I, alternariol monomethyl ether (AME), ansamitocin P3, alternariol (AOH), apicidin, bafilomycin A1, bafilomycin B1, beauvericin, brefeldin A, cytochalasin A, cytochalasin B, cytochalasin C, cytochalasin D, cytochalasin E, cytochalasin H, cytochalasin J, cerulenin, chaetoglobusin A, chaetocin, curvularin, citreoviridin, 10,11–dehydrocurvularin, deoxynivalenol-3-glucoside, deoxynivalenol (DON), destruxin A, destruxin B, dihydrochalasin B, enniatin A, enniatin A1, enniatin B, enniatin B1, fusaric acid, fumonisin B1 (FB1), fumonisin B2, fumonisin B3, fusaric acid, fusarenon-X, gliotoxin, helvolic acid (fumagicin), HT-2 toxin, meleagrin, myriocin, neosolaniol, ochratoxin A (OTA), ochratoxin B, penicillic acid, penitrem A, paxilline, sterigmatocystin, wortmannin, roquefortine-C (ROQ-C), stachybotrylam, T-2 toxin, T-2 triol, T-2 tetraol, tentoxin (TNX), verruculogen, and zearalenone (ZEN). The samples were prepared using a modified QuEChERS method. HPLC-TOF-HRMS analysis was performed on an UltiMate 3000 (Thermo Fisher Scientific, Rochester, NY, USA) HPLC system coupled to a Compact Q-ToF time-of-flight mass spectrometer (Bruker, Germany). Chromatographic separation was performed on a reversed-phase analytical column (Kinetex C_18_, 1.7 µm, 100 Å, 50 × 3.00 mm; Phenomenex, Torrance, CA, USA) at a 0.35 mL min^-1^ flow rate. The analysis was performed in positive full scan mode for all mycotoxins over the *m/z* scanning range from 50 to 1000. The mass extraction window applied for quantification purposes was set to ± 5 ppm at 10,000 full with at half maximum (FWHM) resolution. Data acquisition was controlled by HyStar 3.2. software (Bruker Daltonik GmbH, Bremen, Germany), and data analysis was performed with QuantAnalysis 4.3. software (Bruker Daltonik GmbH, Bremen, Germany).

### 2.8. Evaluation of Biogenic amines (BAs) Formation in Press Cake Samples

Sample preparation and determination of BAs in processing byproduct samples were performed according to Ben-Gigirey et al. [30] with some modifications, which are described by Bartkiene et al. [11].

### 2.9. Analysis of Macro- and Micro-Elements in Processing Byproducts Using Inductively Coupled Plasma Mass Spectrometry (ICP-MS)

The samples were homogenized until the final particle size reached ≤150 µm. Agilent 7700× ICP-MS (Agilent Technologies, Tokyo, Japan) and Mass Hunter Workstation software for ICP-MS, version B.01.03 (Agilent Technologies, Tokyo, Japan) were used for the analysis. Method of macro- and micro-elements analysis in details is described by Bartkiene et al. [31].

### 2.10. Statistical Analysis

All analyses were carried out in triplicate. In order to evaluate the influence of different treatment methods (ultrasonication and fermentation) and their combination on the parameters of processing byproducts the data were subjected to analysis of variance (ANOVA) and the Tukey HSD test as a post-hoc test. The results were considered statistically significant at *p* < 0.05.

## 3. Results and Discussion

### 3.1. Acidity Parameters of Samples after Fermentation

It is known that fermentation reduces pH of fermentable substrate, which leads to higher biosafety of the fermented products. However, it should be mentioned that, apart from the main acidity parameters (pH and TTA), it is very important to evaluate L(+)/D(−) lactic acid isomers ratio, as desirable isomer in food/feed is L-lactic acid, but D-lactic acid can be harmful for mammals [32,33]. The values of pH, TTA, as well as the concentrations of L−(+) and D−(−)-lactic acid in press cakes obtained from rice (RPC), soy (SPC), almonds (APC), coconuts (CPC), and oats (OPC) are given in Table 1. The pH values after 12 h of fermentation ranged from 2.41 to 5.27 for RPC, OPC, and CPC. After 24 h, the pH values of the majority of PC samples increased, except for CPC, in which case the pH value decreased by 0.8. After 48 h of fermentation, the trends were similar as after 24 h, and the lowest pH value was observed for the SPC samples (4.1 ± 0.01), while the highest pH occurred in the OPC samples (8.3 ± 0.02). A strong negative correlation was established between the pH and TTA values (*r* = −0.7019). The highest TTA values were found in SPC samples after 12 h of fermentation (5.5 ± 0.04 °N). In comparison, after 24 h and 48 h of fermentation, the TTA values decreased in all of the treatments (RPC by 8.15%, SPC by 6.20%, APC by 11.20%, CPC by 20.24%, and OPC by 11.03%). The analysis of variance (ANOVA) test showed that both acidity parameters (pH and TTA) were significantly (*p* < 0.05) affected by the selection of the fermentable substrate, duration of fermentation, and the interaction of the analyzed factors (substrate x duration). The acidification of the fermentable substrate is caused by organic acids production that is usually higher in fermentations using LAB starter cultures [34]. However, the alkaline-producing activities also occur during fermentation. The increase in pH during SPC, OPC, RPC, and APC fermentation could be related with the higher protein content in substrate, proteolytic activities, and the release of ammonia by microorganisms involved in fermentation [35]. The same pH tendency was also observed during soybeans and tamarind fermentation [35,36]. The versatile carbohydrate metabolism of LUHS210 strain was reported in previous studies [11,12] and, guided by the acidity parameters, 24 h fermentation was selected as the most effective duration for desirable pH and the TTA values. In all of the fermented samples, higher concentration of L−(+)−lactic acid was observed, compared to the D−(−)−isomer, and the highest L−(+)/D−(−) lactic acid ratio (12.4) was found in APC samples after 24 h fermentation. Normal, lactic acid production in the human body per day is 20–25 mmol/kg of body weight [37]. Lactic acid is produced from pyruvic acid: by the action of L-lactate dehydrogenase for L(+) isomer production, and by the action of D-lactate dehydrogenase for D(−) isomer synthesis. Mammal body cells exclusively synthesize L-lactic acid since their only contain L-lactate dehydrogenase. However, in digestive tract bacteria, which metabolized carbohydrates, can be able to produce D-lactic acid [32]. Mammals can metabolize high concentrations of L-lactic acid; however, D-isomer can accumulate because it is not degraded and can lead to acidosis [37]. Therefore, D-isomer may form in blood and cause acidosis [32]. Finally, a higher amount of L−(+)−lactic acid was produced in the different processed byproducts, compared to D−(−)−lactic acid; thus, the LUHS210 strain can be recommended for the fermentation of RPC, SPC, APC, CPC, and RPC.

### 3.2. Microbiological Parameters of Ultrasonicated and Fermented Processing Byproducts

Total bacteria count (TBC), total enterobacteria count (TEC), lactic acid bacteria (LAB), and mold/yeast (M/Y) in samples after fermentation (12, 24, and 48 h) and ultrasonication are given in Table 2. To ensure favorable microbiological profile and the biosafety of the tested samples, the longer fermentation time was tested. In fact, other metabolites such as enzymes, bacteriocins, that are excreted in later stages of fermentation could influence the reduction of contamination in fermentable substrates. All of the untreated press cake samples showed M/Y contamination, while enterobacteria were found in APC samples (3.08 log_10_ CFU/g). However, ultrasonicated or fermented APC did not contain enterobacteria. TBC and M/Y were not detected in OPC, RPC, CPC, and SPC samples after ultrasonication. Comparison of fermented samples showed that none of these samples contained enterobacteria and M/Y, while the LAB count after 12 h of fermentation ranged from 7.90 log_10_ CFU/g to 8.95 log_10_ CFU/g for CPC and APC, after 24 h of fermentation ranged from 8.38 log_10_ CFU/g to 8.75 log_10_ CFU/g for OPC and APC, and after 48 h of fermentation ranged from 8.32 log_10_ CFU/g to 8.76 log_10_ CFU/g for OPC and RPC. Moderate negative correlation between the pH values of press cake samples and TBC was found (*r* = −0.4103), as well as a weak negative correlation was observed between the pH values of press cake samples and the LAB count (*r* = −0.1082). It was found a significant robust positive correlation between TBC and LAB counts (*r* = 0.906). Also, the method of the treatment, the type of press cake, and their interaction were significant factors (*p* ≤ 0.0001) influenced TBC and LAB counts. The results obtained in this study can be explained by the different mechanisms of microbial inactivation during the fermentation with LAB and ultrasonication. The antimicrobial properties of LAB are explained by the variety of complex compounds, which are produced in fermentable substrate and possess antimicrobial properties by itself [38,39]. The *Lactobacillus* group produces many antimicrobial compounds, and the main ones are lactic and acetic acids. Excreted to environment organic acids reduce the pH of the environment and inhibit a wide spectrum of pathogenic and opportunistic microorganisms. Since microorganisms require a specific pH range for viability and growth, the presence of organic acids produced by LAB limits the growth of pathogenic bacteria [40]. Other antimicrobial compounds that are produced by LAB in much smaller amounts are also very important (e.g., ethanol, formic and free fatty acids, hydrogen peroxide, diacetyl, acetoin, 2,3-butanediol, acetaldehyde, ammonia, benzoate, bacteriocins etc.) [12,41]. Our previous studies showed that the excellent antimicrobial activity was observed for *L. casei* LUHS210 and *L. uvarum* LUHS245, as these strains inhibited all of the opportunistic pathogenic strains tested [12]. Also, it has been demonstrated that some strains of *Lactobacillus casei* excrete acetoin to fermentable environment [42]. The use of ultrasonication for deactivation of microorganisms is one of the non-thermal food processing techniques, since deactivation occurs without heating the bulk food material to high temperatures. Although it has been claimed that ultrasonication alone cannot deactivate bacteria growth, there are other reports in conflicting about deactivation [43]. The relative stiffness and thickness of microorganism cell walls are considered to be crucial factors for the effectiveness of ultrasonication. Bacteria with thin, stiff cell walls are more susceptible to critical failure when subjected to ultrasonic vibration, whereas those comprising thicker, more flexible cell wall materials can dampen the cavitation effects [44]. This study showed that ultrasonication at 37 kHz and fermentation were both effective for improving the biosafety of press cake samples.

### 3.3. The Influence of Ultrasonication and Fermentation on Mycotoxins in Press Cake Samples

**Figure 1:**
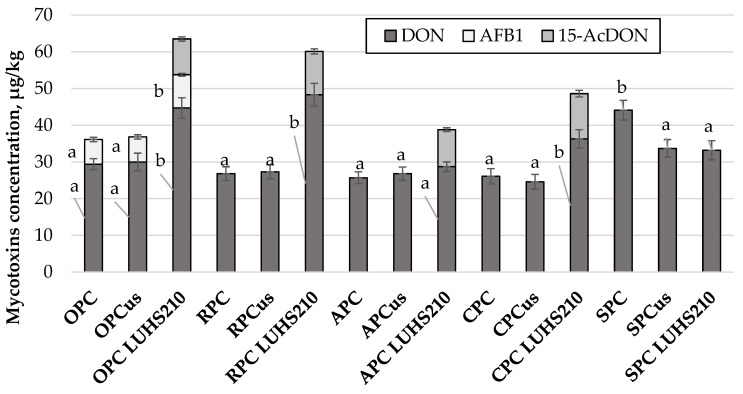
Mycotoxins concentration (µg/kg) in ultrasound treated and fermented with LUHS210 strain byproducts. Data are represented as means (*n* = 3) ± SD. ^a–b^ Means with different letters for same byproduct group are significantly different (*p* < 0.05). RPC—rice press cake; SPC—soy press cake; APC—almond press cake; CPC—coconut press cake; OPC—oat press cake; US—treated with 37 kHz ultrasound; LUHS210—fermented with LUHS210 strain for 24 h; DON—deoxynivalenol; AFB1—aflatoxin B1; 15-AcDON—15-Acetyldeoxynivalenol.

The concentration of mycotoxins in press cake samples that were ultrasonicated or fermented with LUHS210 strain is given in Figure 1. When comparing all of the analyzed samples, the presence of deoxynivalenol (DON) was found in all of the press cake samples, while AFB1 was detected in three press cake samples, and 15-acetyldeoxynivalenol (15-AcDON) was found in four out of the 15 press cake samples analyzed. When comparing the concentrations of DON in OPC samples, the significantly higher (*p* < 0.05) concentration (by 52%) was found in fermented samples, compared to untreated OPC. Ultrasonication did not significantly affect the concentration of DON in RPC and CPC samples, however, the concentration of DON in fermented samples was higher by 80% and 39%, respectively, compared to untreated samples. In APC samples, no significant differences of DON concentration were found, and the mean concentration of DON was 27.07 µg/kg. The opposite trends were observed in SPC samples, where ultrasonication and fermentation reduced the concentration of DON by 24% on average. After fermentation of OPC, RPC, APC, and CPC samples, the formation of 15-AcDON was detected, however 15-AcDON was not found in SPC samples.

The presence of mycotoxins in foods can provoke serious problems to human health (endocrine disruption, carcinogenic, mutagenic etc. effects) [45,46,47]. In the last few decades, due to the common occurrence of mycotoxins in raw material, as well as the efforts to valorize possibly contaminated byproducts for use in food/feed stock, mycotoxins have attracted increasing worldwide attraction. The main mycotoxin from B trichothecenes group is DON, and 3-acetyl deoxynivalenol (3-AcDON), 15-AcDON, nivalenol (NIV), and c X (FX) [48] also belong to this group. DON is also classified as a group 3 carcinogen by the International Agency for Research on Cancer [49]. The lowest permissible limit for DON is 200 μg/kg (in cereal based products), and the highest permissible limit is 1750 μg/kg (in cereals: maize, durum wheat, and oats). In addition to the aforementioned mycotoxins, *Fusarium* fungi are known as producers of moniliformin (MON), enniatins (ENs), beauvericin (BEA), and fusaproliferin (FUS), which are called emerging toxins [50]. In this study, ZEN, alternariol, deoxynivalenol-3-glucoside, HT-2 toxin, enniatin A, enniatin B, enniatin B1, or T-2 toxin were not found in press cake samples. Acetylated derivatives of DON (masked equivalents of DON) - 3-acetyl-DON (3-AcDON) and 15-AcDON are fungal metabolites, which occur during the biosynthesis of DON and are found together with DON [51,52]. It was observed that the hydrolysis of above-mentioned metabolites, as well as its conjugates in the gut of animals fed with contaminated feed, might release the toxic precursor (DON) [51,53]. If the above-mentioned forms of DON are not detected, the toxic potential of a particular sample can be wrongly established and interpreted [54]. It was indicated that the toxicity of acetylated DON derivatives equals that of DON [55]. However, according to the study published by Pinton et al. [56] 15-AcDON is even more toxic than that DON. Published results from samples collected from 12 European countries showed that 20% of the tested samples has a 15-AcDON, as well as 8% are contained with 3-AcDON [57]. European Food Safety Authority (EFSA) published data about the average concentrations of 3-AcDON and 15-AcDON in grains, which were 12.8 and 48.5 μg/kg, respectively. However, 3-AcDON and 15-Ac-DON was quantified in <5% of the food samples [53].

AFB1 was found only in OPC samples (Figure 1). After ultrasound treatment the concentration of AFB1 was similar to the initial OPC, while in fermented OPC samples a significantly higher concentration of AFB1 was found, compared with the initial samples. *Aspergillus flavus* and *A. parasiticus* are the main fungi species, which produce the aflatoxins and cause significant problems to public health. Many types of aflatoxins can be found in nature, however, as particularly dangerous to mammals are B1, B2, G1, and G2 types. Aflatoxins are associated with significant economic losses, which are reaching 1/4 or more of the world’s food crops production [58]. The consumption of food, in which aflatoxin concentration is 1 mg/kg or higher, can lead to aflatoxicosis, for which adults are more tolerant than that children. It was calculated that during consumption over 1–3 weeks, the amount of 20–120 µg/kg AFB1 is an acutely toxic dose that can be lethal [58]. In this study, the concentrations of AFB1 in the analyzed OPC samples were lower and did not exceed the permissible limits [58]. Conclusively, in this study, the byproducts analyzed contained only low concentrations of mycotoxins, which did not reach toxic levels for humans or animals. However, it should be stated that some of the treatments can lead to the formation of masked mycotoxins, and special attention must be paid to the control of fermentation, which typically helps to minimize the presence of mycotoxins in foods of plant origin, as more toxic masked mycotoxins can be formed during the process [59].

### 3.4. Concentration of Biogenic Amines (BAs) in Press Cake Samples

**Figure 2:**
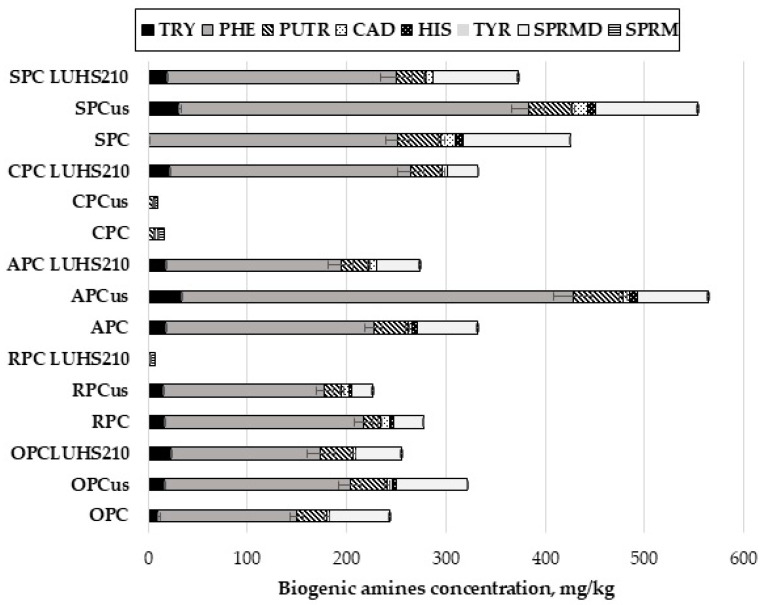
Biogenic amines concentration (mg/kg) in ultrasound treated and fermented with LUHS210 strain byproducts. Data are represented as means (*n* = 3) ± SD. RPC—rice press cake; SPC—soy press cake; APC—almond press cake; CPC—coconut press cake; OPC—oat press cake; US—treated with 37 kHz ultrasound; LUHS210—fermented with LUHS210 strain for 24 h; TRY—tryptamine; PHE—phenylethylamine; PUTR—putrescine; CAD—cadaverine; HIS—histamine; TYR—tyramine; SPRMD—spermidine; SPRM—spermine.

The concentration of BAs in RPC, SPC, APC, CPC, and OPC samples is given in Figure 2. In most of the cases, ultrasonication and fermentation had a significant effect on BAs content in PC samples. When comparing BAs in OPC, the highest concentration of tryptamine (TRY) was found in fermented samples (22.54 ± 1.86 mg/kg), while the concentration in ultrasonicated OPC samples was significantly lower (*p* < 0.05) by 30%, compared to fermented OPC. Phenylethylamine (PHE) was the predominant BA in all press cake samples (except in both initial and ultrasonicated CPC samples), and when comparing the OPC samples, the highest concentration was found in the ultrasonicated samples (187.70 ± 11.02 mg/kg). The average concentration of putrescine (PUT) in OPC samples was 33.59 mg/kg and the average concentration of cadaverine (CAD) was 10 times lower. None of the analyzed fermented press cake samples contained histamine (HIS), and tyramine (TYR) was not detected in untreated and treated PC. Fermentation significantly reduced (*p* < 0.05) the content of spermidine (SPRMD) in OPC, RPC, APC, and SPC samples, while in ultrasonicated RPC, CPC, and SPC samples the content of SPRMD was reduced completely or by 5%–28%. In most cases, fermentation significantly reduced (*p* < 0.05) the concentration of BAs in RPC samples (except for SPRM, which was found at similar concentration after fermentation or ultrasonication). Low concentrations of TRY, PHE, and SPRMD were established in fermented RPC samples, while the other analyzed BAs were not found in these samples. The predominant BA in ultrasonicated RPC samples was PHE and its concentration after ultrasonication significantly decreased (*p* < 0.05) by 19%, compared to untreated RPC. Ultrasonication significantly lowered (*p* < 0.05) the concentration of HIS in RPC samples by 41%. Among the APC samples, ultrasonication increased the content of all BAs, while fermentation reduced the content of BAs, except CAD, compared to untreated samples. When comparing CPC samples, lower content of BAs was found in ultrasonicated samples, compared to the fermented samples. Only three of the eight analyzed BAs were found in untreated samples (PUTR, SPRMD, and SPRM), while samples after ultrasonication contained only two of the eight analyzed BAs (PUTR and SPRM). However, the overall content of BAs in fermented CPC samples was higher by more than 20 times, compared to untreated samples, with PHE as the predominant BA. However, HIS, TYR, and SPRM were not found in fermented CPC samples. When comparing the SPC samples, significantly lower (*p* < 0.05) concentrations of BAs were observed in all of the fermented SPC samples, compared to the untreated or ultrasonicated samples. SPRM was not found in SPC samples, however, higher concentrations of TRY and PHE were found after ultrasonication (by 21% and 40%, respectively). When comparing the total content of BAs in all of the analyzed samples, the highest content was found in APC and SPC samples after ultrasonication (553.8 mg/kg and 564.1 mg/kg). The lowest total BAs content was found in fermented RPC and post- ultrasonication CPC.

Formation of BAs in various substrates is initiated and catalyzed by many factors: physicochemical parameters of substrate, bacterial activity, moisture content, the duration and conditions of storage, as well as the presence in the substrate of free amino acids [60]. The biosynthesis of various BAs strongly depends on the activity of amino acid decarboxylases, which are ubiquitous in microorganisms, however, the biosynthesis and degradation of BAs is also a species-level characteristic of bacteria [61]. It has been reported that *L. casei* 4a and 5b has the ability to promote in vitro degradation of tyramine, histamine, and putrescine [61]. In this study, the analyzed BAs are the most significant contaminants in foods, and each of them has a different precursor, e.g., phenylethylamine can be formed from phenylalanine and spermidine from arginine. It was published about the synergistic effects of BAs toxicity [62,63]. The European Food Safety Authority (EFSA) published a scientific opinion about the risks associated with the BAs formation in fermented products; however, until now, European legislation does not specify threshold concentrations for these compounds [64]. Based on the consumer exposure data, the main toxic BAs in fermented food are HIS and TYR. However, the data is insufficient to evaluate quantitative risk assessment of BAs, individually or synergic action. In fermentable substrate, primary amines are converted into compounds that can be utilized as an energy source, and these changes are initiated by bacteria via oxidative deamination [65]. The potential applications of microorganisms showing amino acid oxidase and dehydrogenase activity has been investigated for the purpose of controlling BAs in foods, with microbial degradation of BAs observed in many cases. It has been published that some bacteria, including certain LAB species, are able to reduce BAs concentration via the action of amino oxidases [66]. From the results obtained in this study, it is clear that fermented press cake samples contained lower total amounts of Bas; however, the formation of BAs is influenced by various factors, and thus, the technology parameters should be optimized for each substrate [26].

### 3.5. Macro- and Micro-Elements in Processing Byproducts after Ultrasonication and Fermentation

**Figure 3:**
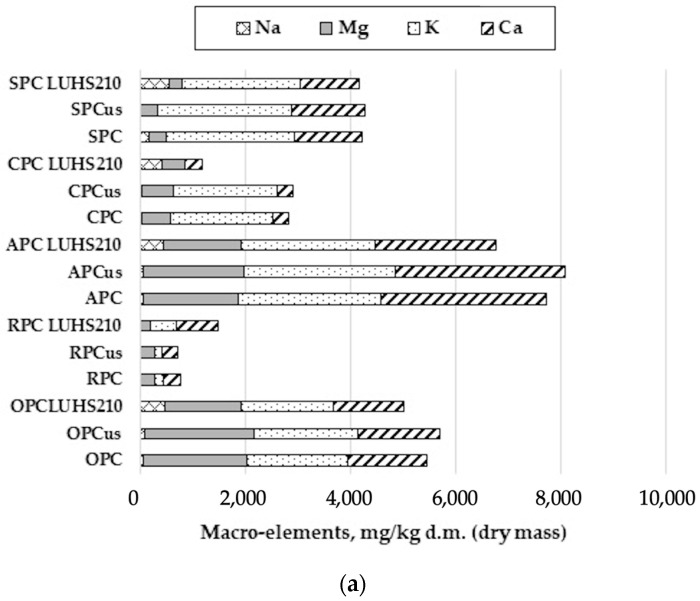

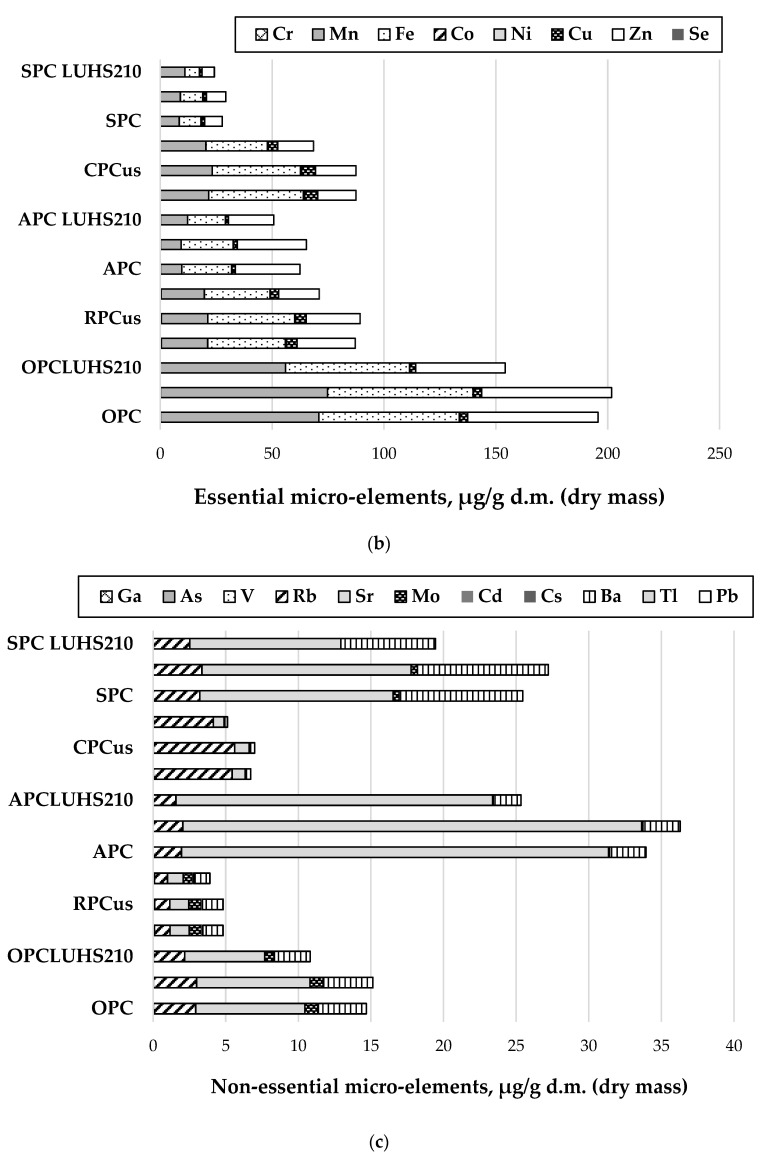
(**a**) Macro-elements, (**b**) essential micro-elements, and (**c**) non-essential micro-elements concentration in ultrasound treated and fermented with LUHS210 strain byproducts. Data are represented as means (*n* = 3) ± SD. RPC—rice press cake; SPC—soy press cake; APC—almond press cake; CPC—coconut press cake; OPC—oat press cake; US—treated with 37 kHz ultrasound; LUHS210—fermented with LUHS210 strain for 24 h.

Minerals are essential inorganic nutrients involved in many physiological functions. Disturbances in mineral metabolism can lead to various health problems [67]. Thus, it is important to study the influence of food processing on the mineral content of various plant matrices, e.g., press cakes. The concentrations of micro- and macro-elements in various press cake samples (RPC, SPC, APC, CPC, and OPC) after ultrasonication and fermentation with the LUHS210 strain are given in Table S1 and Figure 3.

#### 3.5.1. Changes in Macro-Elements

When comparing the content of macro-elements in OPC samples, no significant differences were found among untreated and ultrasonicated samples (Figure 3a). However, when comparing fermented to untreated OPC samples, the concentration of Na was six times higher, and the concentrations of Mg, K, and Ca were lower by 27%, 8%, and 12%, respectively. No significant differences between untreated and ultrasonicated RPC, APC, CPC, and SPC samples were also established. However, significant changes (*p* < 0.05) in the concentrations of macro-elements were found between untreated and fermented samples. Thus, when comparing fermented and untreated RPC samples, the concentrations of Na, K, and Ca in fermented samples were higher by 26.8, 3.2, and 2.3 times, respectively, while the concentration of Mg was lower by 23%. When comparing untreated and fermented APC samples, the concentration of Na in fermented samples was seven times higher, however, the concentrations of Mg, K, and Ca in fermented APC samples was lower by 18%, 6.4%, and 26.6%. In the case of CPC, the concentrations of Na and Ca in fermented samples were 17 times and 8% higher, respectively, however, the concentrations of Mg and K were lower in fermented samples by 23% and 6%, respectively. In the case of SPC samples, fermentation reduced the concentrations of Mg, K, and Ca by 20.4%, 7.8%, and 14.6%, respectively. However, the concentration of Na in fermented samples was almost 3 times higher, compared to the untreated samples.

The macro-elements K, Ca, and Na are structurally important for many proteins, including antioxidant enzymes that protect cells against oxidative stress. Magnesium helps to maintain nerve, muscle, cardiac, and immune function and helps to regulate bone density and blood glucose level, as it is involved in many biochemical processes in the human body [68]. Some minerals, such as Ca and Na, can be entrapped and bonded in complex matrices, which reduce their bioavailability and fermentation usually leads to break these complex molecules (e.g., oxalates and phytate complexes) [69]. The results of Bajwa et al. [70] study showed a significant increase in Mg and Ca content and reduction of K after fermentation of *D. hamiltonii*. The higher mineral content can also be found due to reduction of dry matter during fermentation [71]. However, it has been described in the literature that the content of macro-elements in white sorghum decreased after soaking, cooking, germination, and fermentation processes [72]. It has also been published that the amounts of Ca, as well as P, Fe, and Zn, in sorghum decreased after fermentation [73].

#### 3.5.2. Changes in Essential Micro-Elements

No significant changes were found in essential micro-elements (EM) concentrations comparing the untreated and ultrasonicated samples; however, fermentation influenced most of the EM analyzed (Figure 3b). Selenium was not found in OPC, APC, and SPC samples. When comparing untreated and fermented OPC samples, significantly lower (*p* < 0.05) concentrations of Cr, Mn, Ni, Cu, and Zn were found in the fermented samples. No significant changes of Fe and Co were established between untreated and fermented OPC samples, with the average Fe content equal to 61.07 µg/g and 0.002 µg/g, respectively. When comparing untreated and fermented RPC samples, no significant changes of Cr, Mn, Fe, and Co were found. However, the average concentrations of Ni, Cu, Zn, and Se in fermented RPC samples were significantly lower (*p* < 0.05) compared to untreated samples (by 1.8-, 0.8-, 1.4- and 1.8-fold, respectively). When comparing untreated and fermented APC samples, no significant changes of Cr, Co, and Ni were found. However, the concentration of Mn in fermented APC samples was higher by 27%, in contrast to the concentrations of Fe, Cu, and Zn, which in fermented samples were lower by 24%, 29%, and 30%. Fermentation had no significant influence on the concentrations of Cr, Mn, and Zn in CPC samples, compared to untreated CPC samples. However, the concentrations of Fe, Ni, and Cu in fermented samples were lower by 35%, 38%, and 27%, respectively, in contrast to Co and Se, which increased 2.7-fold and 4.3-fold, respectively, in fermented samples. When comparing all SPC samples, Cr was not found in any of the analyzed SPC samples. Different types of treatment had no significant influence on the concentration of the average concentration of Mn in SPC samples, which was 9.49 µg/g. However, the concentrations of Fe, Co, Ni, Cu, and Zn in fermented samples were significantly lower (*p* < 0.05) compared to the untreated samples.

Iron is an important element involved in a wide range of metabolic processes, including electron transport, deoxyribonucleic acid synthesis, and oxygen transport [74]. A too-low concentration of Fe can lead to anemia, as well as a tiredness and irritability symptoms. Both Fe and Cu are involved in red blood cells formation. Also, Cu is associated with healthy bones, blood vessels, nerves, and immune function, and it also contributes to Fe absorption. It has been reported that fermentation increases the content of Zn and Fe [70]. However, it was also published that fermentation decreases the concentrations of Cu and Mn [70,72]. Ni, Cr, and Co are known to cause allergies in humans. The precise function of Ni in humans is still unknown, but Cr may have an impact on cardiovascular diseases, as well as certain nervous system disorders [75]. The content of these micro-elements in our investigated press cake samples was very low and was further decreased by fermentation. In fermented foods, lactic acid bacteria can accumulate significant quantities of Se, which is considered an essential trace element and plays an important role in human metabolism [76]. In this study, Se was only found in RPC and CPC, and fermentation had a different impact in each case.

#### 3.5.3. Changes in Non-Essential Micro-Elements

The non-essential micro-element Ga was not found in OPC, APC, CPC, and SPC samples (Figure 3c). However, the average concentration of Ga in RPC samples was 0.007 µg/g. Arsenic was also found only in RPC samples, with the average concentration equal to 0.036 µg/g. The non-essential micro-elements V and Cd were not found in APC and SPC samples but were found in OPC, RPC, and CPC samples where the average concentration of V was 0.016, 0.056, and 0.012 µg/g, respectively. The tested types of treatment had no significant effects on the concentration of V. Cadmium was also found in OPC, RPC, and CPC samples, with the average concentration in fermented samples significantly lower (*p* < 0.05) by 31.5%, 23%, and 26%, compared to the untreated samples. None of the analyzed press cake samples contained detectable amounts of Ag, Sn, Sb, and Hg. Titanium was found only in the CPC samples at the average concentration of 0.003 µg/g and its concentration was not significantly affected by various treatment methods. The presence of Pb was detected only in RPC samples at the average concentration of 0.009 µg/g and was not significantly affected by the tested methods of treatment. It was also observed that ultrasonication had no significant effect on the concentrations of Rb, Sr, Mo, and Ba, but significantly lower (*p* < 0.05) average concentrations were found in fermented samples. With regard to the concentration of Cs, it was not found in OPC samples, as well as in fermented SPC samples. Among the other analyzed samples, no significant effects on the concentration of Cs occurred due to the treatment, and the highest content of Cs was found in the CPC samples.

The presence of heavy metals in foods is one of the most important indicators of food safety and quality [77,78,79]. When present in trace quantities, Cu, Zn, Mn, Co, and Mo act as necessary micronutrients, but Cd, As, and Cr are harmful contaminants [80]. Cd and Pb are described as carcinogens [81]. The limit set for Pb are 2 mg/kg and for Cd are 0.1 mg/kg [82]. Cd and Pb toxicity are lower, in comparison with mercury, which is even more toxic and causes a loss of vision, hearing, and mental retardation, and can even be fatal. The permissible level for Hg set by the WHO is 2 µg/kg body weight per day [83] In general, ultrasonication did not significantly influence the content of macro- and micro-elements in press cakes. However, the effect of fermentation with *L. casei* LUHS210 was significant on the concentration of macro- and essential micro-elements in most of the cases. Fermentation also reduced the content of non-essential micro-elements Cd, Rb, Sr, Mo, and Ba in tested press cakes.

## 4. Conclusions

In order to promote the efficient use of press cakes from the production of plant-based beverages, the influence of different physical and biological treatments on the biosafety of press cake should be examined. This study showed that ultrasonication reduced enterobacteria count and M/Y in PC samples, while fermented PC samples did not contain M/Y. All processing byproducts contained DON, but the detected concentrations of mycotoxins were low. Low-frequency ultrasonication and fermentation reduced the average concentration of DON by 24% in the case of soy press cakes. After fermentation, 15-AcDON was formed in all samples except soy press cakes. The lowest total BAs content was found in fermented RPC and post-ultrasonication CPC. Ultrasonication did not significantly affect the concentrations of macro- and micro-elements, while fermentation affected the concentrations of macro-elements and some essential micro-elements in most of the cases. Consequently, both fermentation and ultrasonication are promising tools for enhancing the biosafety of press cake materials. However, the applied treatments can have several effects on BAs, mycotoxins, and elemental nutrients and contaminants in press cakes, and further optimization of such treatments are needed. 

Usually, food preparation is associated with the use of traditional raw materials, to obtain high overall acceptability, desirable structure, quality, safety parameters, and the nutritional value food products. However, food processing should be modified in accordance with population growth, as well as problems associated with the non-desirable climate changes. For this reason, valorization of byproducts has become a crucial issue. In this study, just the first, but the most important, step of the valorization was described, because the safety is the first issue. However, future perspectives for plant-based byproducts valorization are very attractive. Byproducts are beneficial in enrichment and fortification of food products, e.g., they can be used as a source of protein and fiber [84] or a substrate for antifungal sourdough preparation for bread industry; for this purpose, antifungal strains can be applied for byproduct fermentation [85]. As an attractive alternative to the chemical synthesis of vitamins, byproducts can be used as a substrate for biological vitamins production [86]. Finally, byproducts can be used in many industries; however, all safety parameters must meet the given requirements.

## Figures and Tables

**Table 1:** Acidity parameters (pH, total titratable acidity, L−(+) and D−(−) lactic acid concentration) of 12, 24, and 48 h fermented with LUHS210 strain byproducts.

Byproducts	pH			TTA, °N			Lactic Acid Isomers, g/100 g		
	12 h	24 h	48 h	12 h	24 h	48 h	L−(+)	D−(−)	L/D
**RPC**	2.41 ± 0.02	5.4 ± 0.01	5.6 ± 0.01	4.91 ± 0.05	4.17 ± 0.04	3.83 ± 0.03	1.05 ± 0.10	0.31 ± 0.04	3.4
**SPC**	2.88 ± 0.02	3.8 ± 0.02	4.1 ± 0.01	5.5 ± 0.04	4.84 ± 0.05	4.54 ± 0.04	2.13 ± 0.07	0.28 ± 0.03	7.6
**APC**	2.94 ± 0.01	4.6 ± 0.01	4.9 ± 0.02	4.52 ± 0.02	5.09 ± 0.02	4.52 ± 0.02	1.99 ± 0.09	0.16 ± 0.02	12.4
**CPC**	5.27 ± 0.01	4.5 ± 0.02	4.7 ± 0.02	4.45 ± 0.03	4.50 ± 0.03	4.06 ± 0.05	1.87 ± 0.13	0.25 ± 0.04	7.5
**OPC**	2.41 ± 0.02	7.8 ± 0.02	8.3 ± 0.02	4.91 ± 0.06	4.26 ± 0.04	3.79 ± 0.02	1.52 ± 0.10	0.17 ± 0.03	8.9

Data are represented as means (*n* = 3) ± SD. RPC—rice press cake; SPC—soy press cake; APC—almond press cake; CPC—coconut press cake; OPC—oat press cake; TTA—total titratable acidity; L/D—L-(+) and D−(−) lactic acid isomers ratio.

**Table 2:** Microbiological parameters (TBC—total bacteria count; TEC—total enterobacteria count; LAB—lactic acid bacteria; M/Y—mould/yeast) of 12, 24, and 48 h fermented and ultrasound treated byproducts.

Byproducts	TBC	TEC	LAB	M/Y
	log_10_ CFU/g			
OPC	4.02 ± 0.14	nd	nd	3.90 ± 0.18
OPCus	nd	nd	nd	nd
OPC_LUHS210_ (12 h)	8.08 ± 0.14	nd	8.09 ± 0.21	nd
OPC_LUHS210_ (24 h)	8.41 ± 0.25	nd	8.38 ± 0.15	nd
OPC_LUHS210_ (48 h)	8.36 ± 0.21	nd	8.32 ± 0.14	nd
RPC	3.67 ± 0.21	nd	nd	4.64 ± 0.13
RPCus	nd	nd	nd	nd
RPC _LUHS210_ (12 h)	8.34 ± 0.20	nd	8.32 ± 0.18	nd
RPC _LUHS210_ (24 h)	8.75 ± 0.17	nd	8.60 ± 0.20	nd
RPC _LUHS210_ (48 h)	8.80 ± 0.10	nd	8.76 ± 0.20	nd
APC	4.92 ± 0.18	3.08 ± 0.10	nd	4.68 ± 0.17
APCus	4.52 ± 0.11	nd	nd	4.61 ± 0.10
APC _LUHS210_ (12 h)	8.97 ± 0.15	nd	8.95 ± 0.20	nd
APC _LUHS210_ (24 h)	8.81 ± 0.15	nd	8.75 ± 0.17	nd
APC _LUHS210_ (48 h)	8.71 ± 0.17	nd	8.67 ± 0.14	nd
CPC	3.57 ± 0.20	nd	nd	3.23 ± 0.12
CPCus	nd	nd	nd	nd
CPC _LUHS210_ (12 h)	8.27 ± 0.18	nd	7.90 ± 0.15	nd
CPC _LUHS210_ (24 h)	8.32 ± 0.10	nd	8.45 ± 0.23	nd
CPC _LUHS210_ (48 h)	8.42 ± 0.20	nd	8.38 ± 0.16	nd
SPC	5.09 ± 0.15	nd	nd	4.28 ± 0.10
SPCus	nd	nd	nd	nd
SPC _LUHS210_ (12 h)	8.68 ± 0.19	nd	8.52 ± 0.20	nd
SPC _LUHS210_ (24 h)	8.68 ± 0.20	nd	8.70 ± 0.15	nd
SPC _LUHS210_ (48 h)	8.70 ± 0.23	nd	8.61 ± 0.15	nd

Data are represented as means (*n* = 3) ± SD. RPC—rice press cake; SPC—soy press cake; APC—almond press cake; CPC—coconut press cake; OPC—oat press cake; US—treated with 37 kHz ultrasound; LUHS210—fermented with LUHS210 strain for 12, 24, 48 h; TBC—total bacteria count; TEC—total enterobacteria count; LAB—lactic acid bacteria; M/Y—mold/yeast; nd—not found.

## Supplementary Materials

The following are available online at https://www.mdpi.com/2304-8158/9/5/614/s1, Table S1: Micro- and macro-elements concentration in ultrasound treated and fermented with LUHS210 strain byproducts.
