# Correction: Bartkiene et al. Challenges Associated with Byproducts Valorization—Comparison Study of Safety Parameters of Ultrasonicated and Fermented Plant-Based Byproducts. *Foods* 2020, *9*, 614

**DOI:** 10.3390/foods13172737

**Published:** 2024-08-29

**Authors:** Elena Bartkiene, Vadims Bartkevics, Iveta Pugajeva, Anastasija Borisova, Egle Zokaityte, Vita Lele, Vytaute Sakiene, Paulina Zavistanaviciute, Dovile Klupsaite, Daiva Zadeike, Fatih Özogul, Grazina Juodeikiene

**Affiliations:** 1 Institute of Animal Rearing Technologies, Lithuanian University of Health Sciences, Tilzes g. 18, LT-47181 Kaunas, Lithuania; egle.zokaityte@lsmuni.lt (E.Z.); vita.lele@lsmuni.lt (V.L.); vytaute.sakiene@lsmuni.lt (V.S.); paulina.zavistanaviciute@lsmuni.lt (P.Z.); dovile.klupsaite@lsmuni.lt (D.K.); 2 Department of Food Safety and Quality, Lithuanian University of Health Sciences, Tilzes g. 18, LT-47181 Kaunas, Lithuania; 3 Centre of Food Chemistry, University of Latvia, Jelgavas iela 1, LV-1004 Riga, Latvia; vadims.bartkevics@bior.lv; 4 Institute of Food Safety, Animal Health and Environment BIOR, Lejupes iela 3, LV-1076 Riga, Latvia; iveta.pugajeva@bior.lv (I.P.); anastasija.borisova@bior.lv (A.B.); 5 Department of Food Science and Technology, Kaunas University of Technology, Radvilenu Rd. 19, LT-50254 Kaunas, Lithuania; daiva.zadeike@ktu.lt (D.Z.); grazina.juodeikiene@ktu.lt (G.J.); 6 Department of Seafood Processing Technology, The University of Cukurova, Balcali, Saricam, 01330 Adana, Turkey; fozogul@cu.edu.tr

In the original publication [1], there was a mistake in Figures 2 and 3a. In Figure 2, not all names of the samples are visible. In Figure 3a, mg/kg should be used instead of mg/g. The corrected Figure 2 and Figure 3a appear below. The authors state that the scientific conclusions are unaffected. This correction was approved by the Academic Editor. The original publication has also been updated.

## Figures and Tables

**Figure 2:**
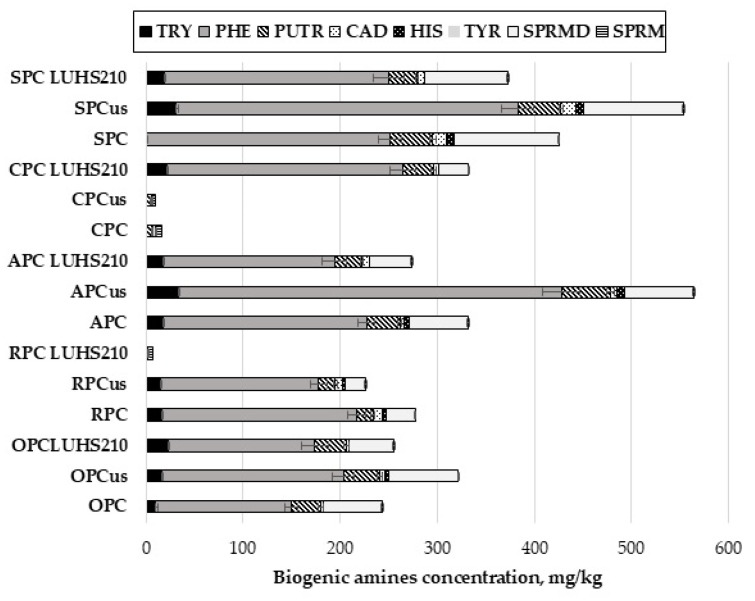
Biogenic amines concentration (mg/kg) in ultrasound treated and fermented with LUHS210 strain byproducts. Data are represented as means (*n* = 3) ± SD. RPC—rice press cake; SPC—soy press cake; APC—almond press cake; CPC—coconut press cake; OPC—oat press cake; US—treated with 37 kHz ultrasound; LUHS210—fermented with LUHS210 strain for 24 h; TRY—tryptamine; PHE—phenylethylamine; PUTR—putrescine; CAD—cadaverine; HIS—histamine; TYR—tyramine; SPRMD—spermidine; SPRM—spermine.

**Figure 3:**
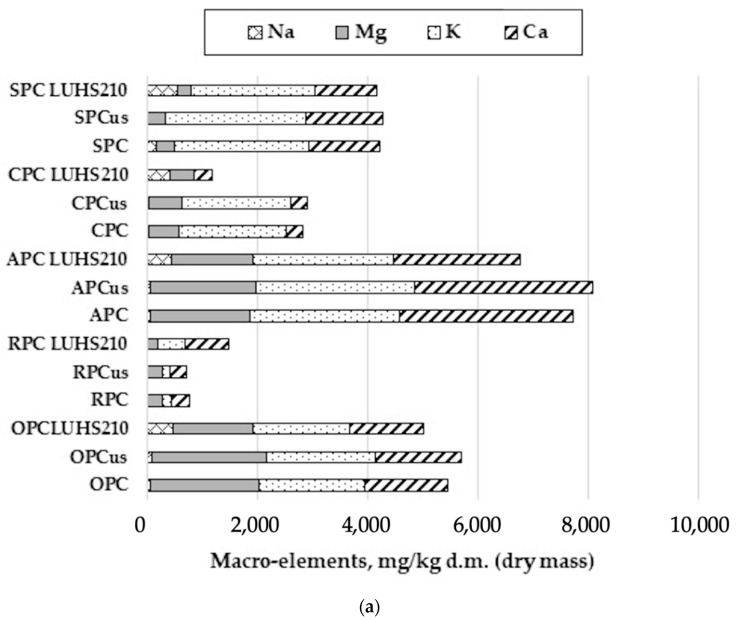

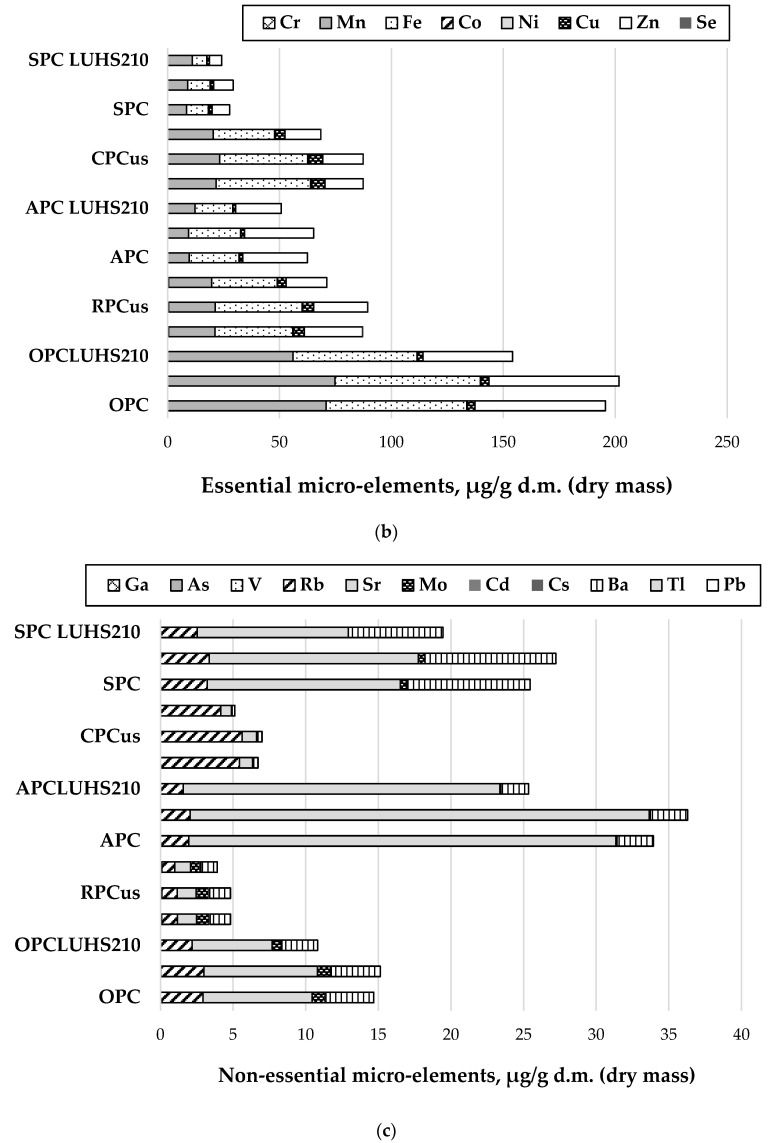
(**a**) Macro-elements, (**b**) essential micro-elements, and (**c**) non-essential micro-elements concentration in ultrasound treated and fermented with LUHS210 strain byproducts. Data are represented as means (*n* = 3) ± SD. RPC—rice press cake; SPC—soy press cake; APC—almond press cake; CPC—coconut press cake; OPC—oat press cake; US—treated with 37 kHz ultrasound; LUHS210—fermented with LUHS210 strain for 24 h.
